# Identification of Main Genetic Causes Responsible for Non-Syndromic Hearing Loss in a Peruvian Population

**DOI:** 10.3390/genes10080581

**Published:** 2019-07-31

**Authors:** Erick Figueroa-Ildefonso, Guney Bademci, Farid Rajabli, Mario Cornejo-Olivas, Ruy Diego Chacón Villanueva, Rodolfo Badillo-Carrillo, Miguel Inca-Martinez, Karina Milla Neyra, Claire Sineni, Mustafa Tekin

**Affiliations:** 1 Neurogenetics Research Center, Instituto Nacional de Ciencias Neurológicas, Lima 15003, Peru; 2 John P. Hussman Institute for Human Genomics, University of Miami Miller School of Medicine, Miami, FL 33136, USA; 3 Dr. John T. Macdonald Foundation Department of Human Genetics, University of Miami Miller School of Medicine, Miami, FL 33136, USA; 4 Center for Global Health, Universidad Peruana Cayetano Heredia, Lima 15102, Peru; 5 Inter-units Program in Biotechnology, Institute of Biomedical Sciences, University of São Paulo, São Paulo 05508-270, Brazil; 6 Centro de Investigaciones Básicas en el Área Otoneurológica, Instituto Nacional de Ciencias Neurológicas, Lima 15003, Peru; 7 Lerner Research Institute, Genomic Medicine, Cleveland Clinic Foundation, Cleveland, OH 44195, USA

**Keywords:** hearing loss, GJB2, non-syndromic, Peruvian

## Abstract

Hearing loss (HL) is a common sensory disorder affecting over 5% of the global population. The etiology underlying HL includes congenital and acquired causes; genetic factors are the main cause in over 50% of congenital cases. Pathogenic variants in the *GJB2* gene are a major cause of congenital non-syndromic hearing loss (NSHL), while their distribution is highly heterogeneous in different populations. To the best of our knowledge, there is no data regarding the genetic etiologies of HL in Peru. In this study, we screened 133 Peruvian families with NSHL living in Lima. We sequenced both exons of the *GJB2* gene for all probands. Seven probands with familial NSHL that remained negative for *GJB2* variants underwent whole genome sequencing (WGS). We identified biallelic pathogenic variants in *GJB2* in 43 probands; seven were heterozygous for only one allele. The c.427C>T variant was the most common pathogenic variant followed by the c.35delG variant. WGS revealed three novel variants in *MYO15A* in two probands, one of them was predicted to affect splicing and the others produce a premature stop codon. The Peruvian population showed a complex profile for genetic variants in the *GJB2* gene, this particular profile might be a consequence of the admixture history in Peru.

## 1. Introduction

Hearing loss (HL) is a common sensory disorder affecting over 5% of the global population [1]. The etiology underlying HL includes congenital and acquired causes; genetic factors are causative in over 50% of congenital cases [2]. The mode of inheritance can be autosomal recessive (~80%), autosomal dominant (~20%), mitochondrial, or X-linked (1%–1.5%) [3]. DNA variants in over 100 genes have been reported to cause non-syndromic hearing loss (NSHL) [4], and their distribution is highly heterogeneous in different populations [5].

Pathogenic variants in the *GJB2* gene (NM_004004.6) are considered to be the major cause of congenital NSHL across populations [6]. The *GJB2* gene has two exons; the second exon codes for the gap junction beta-2 (or Cx26) protein, involved in the maintenance of a high endocochlear potential [7]. The pathogenic variant c.35delG has been reported as the most common genetic cause of NSHL with European ancestry [8]. In populations from East Asia, such as Japan, China, and Korea, the c.235delC variant is the most common mutation [9,10,11,12]. In Latin American populations, such as Argentina and Mexico, c.35delG is the most common variant [13,14]. Around 1.8% of the Peruvian population have significant HL, of whom up to 11% of cases are congenital [15]. To the best of our knowledge, there is no data regarding genetic etiologies of HL in Peru.

Peru is a South American country hosting an admixed population with a significant Amerindian component. Due to many historical events, including migration before and during the Inca Empire and Spanish colonization, the global ethnic structure of the Peruvian population comprises ~80% of Amerindian, 7.4% of European, 4.4% of Oceanian, 3.5% of East Asian, and 1.7% of African ancestries. However, distribution varies across regions within Peru [16,17].

In this study, we screened 133 Peruvian families with NSHL living in Lima. We sequenced the two exons of the *GJB2* gene for all probands. Seven probands from multiplex families, negative for *GJB2* variants, underwent whole genome sequencing (WGS). In addition, we present ancestry analysis performed for the most common pathogenic variant (*GJB2* NM_004004.6; c.427C>T) in order to determine the possible origin(s) of this variant in Peru.

## 2. Methods

### 2.1. Subjects

DNA samples from 133 unrelated probands (63 multiplex and 70 simplex cases) affected with NSHL were obtained from the biorepository of the Neurogenetics Research Center (NRC) at the *Instituto Nacional de Ciencias Neurológicas* (INCN), from Lima, Peru, collected for a previous study. Diagnosis of all probands with NSHL was based on clinical and audiology assessment performed by an otolaryngologist. All cases were screened for environmental factors through a survey given to the mothers of the cases. The survey queried prenatal, natal, and postnatal infections, aminoglycoside exposure, prematurity, perinatal hypoxia, need for neonatal intensive care, and head and sound trauma, for which all cases were negative. All DNA samples were negative for two *GJB6* deletions by multiplex PCR from a previous study [18].

This study was approved by the Ethics Committee of the *Instituto Nacional de Ciencias Neurológicas* (INCN) and the IRB of the University of Miami.

### 2.2. Sanger Sequencing of GJB2 Exon 1 and Exon 2

All samples were analyzed at the Hussman Institute for Human Genomics at the University of Miami. We performed Sanger sequencing of both exons of the *GJB2* gene using primers 5’-CCGGGAAGCTCTGAGGAC-3’, 5’-GCAACCGCTCTGGGTCTC-3’ for exon 1; and 5’-TTGGTGTTTGCTCAGGAAGA-3’, 5’-GGCCTACAGGGGTTTCAAAT-3’ for exon 2. PCR conditions were the same as previously described [19]. Visualization of PCR products was on agarose gels followed by cleaning over Sephadex columns according to the manufacturer’s recommendations. We performed sequence analysis using the Big Dye Terminator v3.1 Cycle Sequencing Kit and the ABI PRISM 3130 Genetic Analyzer (Applied Biosystems, Foster City, USA). Sequence traces were analyzed using the Sequencher^®^ 5.0 program (Gene Codes Corporation, Ann Arbor, USA). Classification of genetic variants was performed following the American College of Medical Genetics (ACMG) guidelines [20].

### 2.3. Whole Genome Sequencing Analysis

Seven unrelated probands from multiplex families, negative for any pathogenic or likely pathogenic variant in *GJB2*, were subjected to WGS using a BGISEQ-500 instrument with paired-end 100 bp protocol [21]. Reads were mapped to the human reference genome (NCBI build37/hg 19 version). Burrows-Wheeler Aligner (BWA) software was used for alignment and the Genome Analysis Toolkit (GATK) was used for variant calling [22,23]. Copy number variants (CNVs) were called using the CNVnator software [24]. Structural variations (SV) were screened with Breakdancer [25].

Analysis of WGS data was limited to NSHL genes listed on the Hereditary Hearing Loss Homepage [4]. Only exons and up to 20 bp flanking intronic regions were analyzed in these genes. Minor allele frequency thresholds of 0.005 for recessive and 0.0005 for dominant variants were considered by using the gnomAD (http://gnomad.broadinstitute.org/) and dbSNP (https://www.ncbi.nlm.nih.gov/projects /SNP/) databases, as well as the HIHG internal WES/WGS database that includes >4000 exomes/genomes from different ethnicities. We also filtered variants by using the combination criteria of damaging for SIFT (http://sift.jcvi.org/), disease-causing for MutationTaster (http://www.mutationtaster.org/), CADD score >20 (https://cadd.gs.washington.edu/) and GERP score >2 (http://mendel.stanford.edu/SidowLab/downloads/gerp/). Sanger sequencing was used to confirm detected variants.

### 2.4. Genome-Wide Genotyping and Quality Control

Genome-wide genotyping was performed by using the Global Screening Array (GSA) v1 (Illumina, San Diego, CA, USA). We assessed the quality control using the PLINK software, v.2 [26]. Firstly, we excluded samples with a low call rate (>90%), and markers with a genotype missingness of 3%, and Hardy–Weinberg equilibrium departure (*p* < 10^−6^) and minor allele frequency less than 0.05. Then, we applied the linkage disequilibrium pruning method at pairwise r^2^ < 0.4 to select the tagging SNPs from the array dataset.

### 2.5. Admixture Analysis

The Peruvian population is an admixture of four parental ancestries: Native Americans (AI), European (EU), West African (AF), and East Asian (EA). To estimate the proportion of the parental ancestries within Peruvians, we used a model-based clustering algorithm implemented in the ADMIXTURE software [27]. We performed supervised ADMIXTURE analysis at K = 4 by including the reference panels (AI, EU, AF, and EA populations) from the Human Genome Diversity Project [28].

### 2.6. Local Ancestry Analysis

We used the same reference panel (selected for global ancestry analysis) to calculate the local ancestral components of chromosome 13 in 20 unrelated probands (1 homozygous and 19 heterozygous) positive for the c.427C>T variant. We assessed local ancestry using a reference panel from three continental populations (AI, EU, and AF). We first phased the reference panel with the Peruvian dataset by using the Segmented Haplotype Estimation and Imputation tool ver. 2 (ShapeIT) [29]. Then, we used RFMix [30], a discriminative modeling approach, to infer the local ancestry across chromosome 13. To improve the accuracy of haplotypes, we ran the RFMix with the PopPhased option in a minimum node size 5. Then, we defined a 2 Mb region (chr13:19,000,000–21,000,000) that stretched the local ancestral block around the c.427C>T variant [31]. The region included 25 variants common in both the reference panel and the Peruvian genotyped dataset (Table S1).

## 3. Results

Hearing loss in all cases had a prelingual or congenital onset (average age of onset was 18.2 months), with severity ranging from moderate to profound. Around 52% of probands described here were males and 48% were females (Table S2). Lima was the city of birth in 81.2% of cases.

### 3.1. GJB2 Sequencing

Among the 133 analyzed cases, 43 (32%) had biallelic pathogenic variants in *GJB2* and seven were heterozygous for only one allele (Tables S3 and S4). The overall allele frequency of pathogenic variants in *GJB2* was 35% (93/266). We found eight pathogenic and six likely pathogenic *GJB2* variants. The c.427C>T variant, causing a change in codon 143 from an arginine to a tryptophan p.(Arg143Trp), was identified in 20 probands (19 heterozygous and 1 homozygous), representing 22.6% (21 out of 93) of alleles. The c.35delG variant was the second most common variant, identified in 17.2% of mutated alleles. Remarkably, we identified six heterozygous probands for the rarely reported pathogenic variant c.645delT (Table 1). In addition, we identified three benign variants and four variants of uncertain significance (VUS) (Table S3). Benign variants were found with a pathogenic variant or alone. Only the VUS c.154G>T p.(Val52Phe) was found together with a likely pathogenic variant, c.94C>A (p.Arg32Ser).

### 3.2. Ancestry Analysis

**Figure 1:**
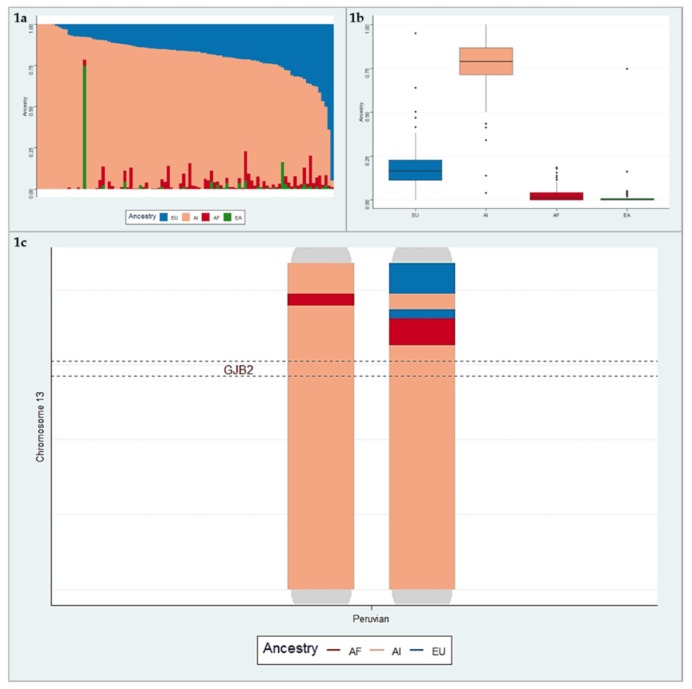
**a.** Bar-plot of four-way admixed Peruvian individuals estimated using ADMIXTURE software at K = 4. **b.** The box-plot of the average ancestries in Peruvian individuals. **c.** Illustration of local ancestry on chromosome 13. This figure represents admixture blocks “local ancestry”, with each ancestry coded by a different color (red: African (AF), blue: European (EU), pink: American Indian (AI)), from a Peruvian individual.

We examined the population structure of Peruvians using the supervised ADMIXTURE analysis at K = 4. Figure 1a illustrates the results from the ADMIXTURE analysis in a bar-plot figure. Each vertical bar represents an individual, which shows an estimate of the fraction of continental ancestries (AI, AF, EU, and EA). On average, the fraction of AI ancestry in Peruvians was the highest with the mean 76.9% (SD = 16.9). The EU, AF, and EA ancestry proportions were split, with the mean values 18.7% (SD = 13.8), 2.9% (SD = 4), and 1.5% (SD = 7.8), respectively (Figure 1b). Results of the admixture analysis agree with the recent genetic studies showing a four-way admixture (AI, EU, AF, and EA) structure in Peruvians.

### 3.3. Haplotype Analysis for Variant c.427C>T 

**Figure 2:**
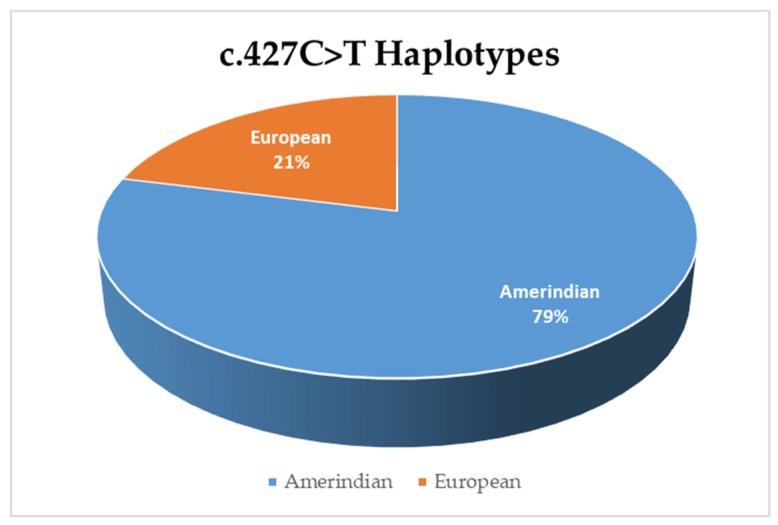
Frequency of the haplotypes in cases positive for the c.427C>T variant.

We found that Peruvian cases have two haplotypes for the c.427C>T variant, a European and an Amerindian haplotype. The Amerindian haplotype is present in 79% of cases carrying the c.427C>T (Figure 2). The African haplotype is absent in the studied sample. 

### 3.4. Whole Genome Sequencing

We did not detect pathogenic or likely pathogenic variants (single nucleotide, indel, or copy number variants) in known NSHL genes in five probands. We found three causative variants within the *MYO15A* gene (NM_016239.3) in the remaining two probands. One proband was homozygous for the c.3757-2A>G variant, located in a canonical splice site two base pairs upstream of exon 5. The second proband was heterozygous for two variants: c.843C>A, which is a nonsense variant in exon 2, and c.8798delT, which produces a frameshift in the open reading frame of exon 50 (Table S5). These genetic variants have not been reported in the public databases gnomAD or 1000 genomes project. 

## 4. Discussion

This is the first study where a Peruvian population with NSHL was screened for genetic variants in the *GJB2* gene. We found 14 causative variants in 50 cases among 133 probands in a Peruvian sample affected with NSHL. The most frequent variant was c.427C>T; haplotype analysis suggests both Amerindian and European origin for this variant. Among the seven probands that completed WGS, we found three novel causative variants in *MYO15A*.

Our Peruvian cohort presents a complex profile for *GJB2* variants, with two pathogenic variants being the most frequent ones. The c.427C>T variant was the most common of the pathogenic variants (22.6%). It has been reported as the main genetic cause of HL in Ghana [32]. In Japanese and Korean populations, the c.427C>T variant was reported as the second most common pathogenic variant with a frequency of 21.4% and 26.8% among 70 and 41 mutated alleles, respectively [12,33]. 

In contrast, studies in Latin-American populations reported that c.427C>T is an infrequent cause of HL. In an Argentinian study, the c.427C>T variant was reported as the second most common pathogenic variant after c.35delG, with only 4 of 68 mutated alleles [14]. In Ecuador, this variant was present in three alleles from a total of 100 mutated alleles [34]. In Mexican and Brazilian populations, this variant is almost absent, reported only once in 78 Mexican subjects [13,35,36] and in 1 in 74 Brazilian cases [37]. On the other hand, this variant was not reported in Colombian and Venezuelan populations where *GJB2* was sequenced [38,39]. In this regard, the high frequency of c.427C>T found in Peruvian subjects contrasts with other Latin-American populations, where c.35delG is reported as the most common variant and c.427C>T has a low frequency. Although none of these studies included admixture analysis, the difference in allelic frequencies might be explained by a different ancestral composition of populations in each study. We cannot discard biases due to sample size and lack of representability of the population.

The cohort analyzed in this study was mainly born in Lima and showed an Amerindian composition of 76.9%, which is higher than the average for a Peruvian population from Lima. The high percent of Amerindians might explain the high frequency of an Amerindian haplotype in Peruvian cases carrying the c.427C>T variant. These findings are remarkable as the c.427C>T variant is the most frequent causative *GJB2* variant in the African population of Ghana and relatively common in East Asians. Our haplotype analysis suggests that the most common deafness variant in Peru has multiple origins, perhaps one in America and another that has migrated from Europe. Alternatively, this variant may have a single ancient origin and was brought to the Americas twice: first during the migration from the Behring strait thousands of years ago and subsequently by Europeans five centuries ago.

Interestingly, six probands (five multiplex and one simplex cases) were heterozygous for the rare variant c.645delT. Clinical data available for three of these cases showed profound HL. This variant was previously reported in two studies from the U.S., and was identified in only one case among 119 and 384 probands with severe to profound HL, respectively. The authors did not specify the ethnic origin of these subjects [40,41]. While more studies are needed to assess the origin of c.645delT, its presence in six probands suggests a founder effect in this population.

We identified three novel variants in *MYO15A* in two of seven *GJB2* negative cases (Table S5). There are more than 150 reported pathogenic and likely pathogenic variants in *MYO15A*, which is considered to be a relatively common gene for recessive NSHL [42,43]. The c.3757-2A>G variant is predicted to most likely affect splicing (www.umd.be/HSF/) within the splice acceptor region located 2 bp upstream of exon 5. The c.843C>A variant is located in exon 2 and causes a premature stop codon. The c.8798delT variant causes a frameshift in exon 50, leading to a premature stop codon. In silico analysis shows that both variants likely trigger nonsense mediated decay with a loss of 3250 and 498 amino acids, respectively (http://www.mutationtaster.org/). Our findings suggest that *MYO15A* variants are relatively common, following those in *GJB*2, in the Peruvian population, which should be considered in further mutation screenings. In addition, the absence of causative variants in known deafness genes in five multiplex cases suggests the existence of novel genes to be discovered in the Peruvian population.

This is the first report of the genetics of HL in a Peruvian cohort; the results show new and remarkable findings in comparison to other Latin-American populations. However, some limitations should be noted. First, the samples included in this study are not truly representative of Peru as all samples were collected in Lima, and ~80% of subjects were born in Lima. In addition, we did not have access to parental samples to confirm compound heterozygosity.

## 5. Conclusions

The Peruvian population shows a complex profile for genetic variants in the *GJB2* gene, which is likely caused by the admixed composition of the Peruvian cohort. The dual origin, Amerindian and European, of the c.427C>T in the Peruvian sample suggests that HL mutations in Peru have heterogeneous origins throughout history.

## Figures and Tables

**Table 1:** Pathogenic variants identified in *GJB2.*

Pathogenic Variant	Heterozygous	Homozygous	Total of Families	Frequency *
c.427C>T; p.(Arg143Trp)	19	1	20	22.6%
c.35delG; p.(Gly12fs)	10	3	13	17.2%
**c.94C>A; p.(Arg32Ser)**	9	1	10	11.8%
c.59T>C; p.(Ile20Thr)	8	1	9	10.8%
c.35G>T; p.(Gly12Val)	3	3	6	9.7%
c.283G>A; p.(Val95Met)	6	0	6	6.5%
c.645delT; p.(Arg216fs)	6	0	6	6.5%
c.19C>T; p.(Gln7Ter)	5	0	5	5.4%
c.167delT; p.(Leu56fs)	3	0	3	3.2%
c.109G>A; p.(Val37Ile)	2	0	2	2.2%
c.29T>C; p.(Leu10Pro)	1	0	1	1.1%
c.235delC; p.(Leu79fs)	1	0	1	1.1%
**c.617A>G; p.(Asn206Ser)**	1	0	1	1.1%
c.232dupG; p.(Ala78fs)	1	0	1	1.1%

* Allele frequencies calculated among individuals with pathogenic and likely pathogenic variants.

## Supplementary Materials

The following are available online at https://www.mdpi.com/2073-4425/10/8/581/s1, Table S1: List of SNPs used for haplotype construction, Table S2: Summary of clinical features of cases, Table S3: Characteristics of identified GJB2 variants, Table S4: List of *GJB2* genotypes, Table S5: Causative genetic variants identified via WGS.
