# Supplementary material for: Identification of Main Genetic Causes Responsible for Non-Syndromic Hearing Loss in a Peruvian Population
Source: Genes (Basel). 2019 Jul 31;10(8):581. doi: 10.3390/genes10080581 (PMC6723399; doi:10.3390/genes10080581)
Supplement: Supplementary file 1 [file genes-10-00581-s001.pdf]

Supplementary Materials:

Table S1. List of SNPs used for haplotype construction.

RS id	Position in chromosome 13 (Build GRCh37)
rs7984141	19,660,641
rs9578792	19,662,154
rs4769387	19,666,481
rs7320243	19,714,717
rs518637	19,732,341
rs11617480	20,211,434
rs7988277	20,411,701
rs6490494	20,493,020
rs7327207	20,494,558
rs9506408	20,507,957
rs1407961	20,572,751
rs4624004	20,684,742
rs2031283	20,687,836
rs7982556	20,688,590
rs1011038	20,695,554
rs1886176	20,715,801
rs9509057	20,723,127
rs7981756	20,790,742
rs7992230	20,848,317
rs2050500	20,854,763
rs1953516	20,881,146
rs1572371	20,887,860
rs4409941	20,951,425
rs4331204	20,962,029
rs4770024	20,995,731

Table S2. Summary of clinical features of cases.

Clinical features	
**Age at onset**	
Mostly prelingual	18.2 months [0 – 72 m]
**Sex**	
Males	69
Females	64
**Family history**	
Multiplexcases	63
Simplex cases	70
**Level hearing loss on left ear**	
Moderate	2.9%
Severe	14.7%
Profound	82.4%
**Level hearing loss on right ear**	
Moderate	1%

Severe	13.7%
Profound	85.3

*Complete audiometry information was available for 102 cases. Grades of hearing loss according to WHO: Mild (26 – 40 dB), Moderate (41 – 60 dB), Severe (61 – 80 dB), Profound (Over 81 dB).

TableS3. Characteristics of identified *GJB2* variants.

cDNA	Protein	SIFT	Mutation Taster	GERP[1]	PhastCons[1]	gnomAD	Reference	ClinVar	ACMG
c.19C>T	p.(Gln7Ter)	NA	DC	5.36	0.023	0.00000802	Pandya et al. 2003	Pathogenic/Likely pathogenic	Pathogenic
c.29T>C	p.(Leu10Pro)	DM	DC	5.36	0.335	-	Dalamón et al. 2013	NA	Likely pathogenic
c.35G>T	p.(Gly12Val)	0.001/DM	DC	5.36	0.591	0.00009221	Rabionet, R. et al. 2000	Pathogenic/Likely pathogenic	Pathogenic
c.35delG	p.(Gly12fs)	NA	NA	5.36	NA	0.006188	Zelanmte, L. et al. 1997	Pathogenic	Pathogenic
c.59T>C	p.(Ile20Thr)	0.02/DM	DC	5.21	0.961	-	Loffler, J. et al. 2001	Likely pathogenic	Likely pathogenic
c.94C>A	p.(Arg32Ser)	0/DM	DC	5.21	0.8939	0.000007984	Hayashi C. et al. 2011	Likely pathogenic	Likely pathogenic
c.109G>A	p.(Val37Ile)	T	DC	5.21	0.5149	0.007556	Kelley, P., et al. 1998	Conflicting interpretations of pathogenicity	Likely pathogenic
c.167delT	p.(Leu56fs)	NA	NA	4.1199	NA	0.0008036	Zelante et al. 1997	Pathogenic	Pathogenic
c.232dupG	p.(Ala78fs)	NA	NA	5.3299	NA	NA	Putcha et al 2007	Likely pathogenic	Likely pathogenic
c.235delC	p.(Leu79fs)	NA	NA	5.3299	-	0.0004738	Kudo T et al. 2000	Pathogenic	Pathogenic
c.283G>A	p.(Val95Met)	0.001/DM	DC	4.2399	0.9959	0.00004779	Kelley, P., et al. 1998	Pathogenic/Likely pathogenic	Pathogenic
c.427C>T	p.(Arg143Trp)	0/DM	DC	4.56	0.92	0.0001311	Brobby, G.; Muller-Myhsok, B. 1998	Pathogenic	Pathogenic
c.617A>G	p.(Asn206Ser)	T	DC	4.449	0.448	0.0001027	Marlin et al. 2001	Pathogenic	Pathogenic
c.645delT	p.(Arg216fs)	NA	NA	-2.96	NA	NA	Angeli 2009	Likely pathogenic	Likely pathogenic
c.-15C>T	NA	NA	NA	-5.0799	NA	0.004994	Gasmelseed NM et al. 2004	Benign/Likely benign	Benign
c.79G>A	p.(Val27Ile)	T	POL	5.21	0.898	0.05038	Kelley, P., et al. 1998	Benign	Benign
c.*84T>C	NA	NA	NA	5.82	NA	0.7486	Wilch et al. 2006	Benign	Benign
c.*1C>T	NA	NA	NA	-3.96	NA	0.0004986	Dalamón et al. 2010	Conflicting interpretations of pathogenicity	Uncertain significance
c.-22-6T>C	NA	NA	NA	1.74	NA	0.0004889	Tang et al. 2006	Uncertain significance	Uncertain significance
c.154G>T	p.(Val52Phe)	DM	POL	-7.3499	0.05099	NA	NA	NA	Uncertain significance

c.503A>G	p.(Lys168Arg)	T	DC	5.4699	0.8399	0.0000531	Samanich J et al. 2007	Conflicting interpretations of pathogenicity	Uncertain significance

DM: Damaging, **T**: Tolerated, **DC**: Disease Causing, **NA**: Not Available, **ACMG**: American College of Medical Genetics Guideline (Richards et al., 2015).
[1]PhastCons and GERP scores are obtained from SeattleSeq Annotation 138 (http://snp.gs.washington.edu/SeattleSeqAnnotation138/).

Table S4. List of *GJB2* genotypes.

COMPOUND HETEROZYGOUS GENOTYPES		PROBANDS
c.[427C>T(;)94C>A]	p.[Arg143Trp(;)Arg32Ser]	5
c.[427C>T(;)59T>C]	p.[Arg143Trp(;)Ile20Thr]	4
c.[427C>T(;)35delG]	p.[Arg143Trp(;)Gly12fs]	3
c.[427C>T(;)645delT]	p.[Arg143Trp(;)Arg216fs]	3
c.[427C>T(;)35G>T]	p.[Arg143Trp(;)Gly12Val]	1
c.[427C>T(;)167delT]	p.[Arg143Trp(;)Leu56fs]	1
c.[427C>T(;)109G>A]	p.[Arg143Trp(;)Val37Ile]	1
c.[427C>T(;)427C>T]	p.[Arg143Trp(;)Arg143Trp]	1
c.[427C>T(;)19C>T]	p.[Arg143Trp(;)Gln7Ter]	1
c.[35delG(;)35delG]	p.[Gly12fs(;)Gly12fs]	3
c.[35delG(;)94C>A]	p.[Gly12fs(;)Arg32Ser]	2
c.[35delG(;)19C>T]	p.[Gly12fs(;)Gln7Ter]	2
c.[35delG(;)283G>A]	p.[Gly12fs(;)Val95Met]	1
c.[35G>T(;)35G>T]	p.[Gly12Val(;)Gly12Val]	3
c.[35G>T(;)235delC]	p.[Gly12Val(;)Leu79fs]	1
c.[59T>C(;)283G>A]	p.[Ile20Thr(;)Val95Met]	2
c.[59T>C(;)59T>C]	p.[Ile20Thr(;)Ile20Thr]	1
c.[645delT(;)167delT]	p.[Arg216fs(;)Leu56fs]	1
c.[645delT(;)283G>A]	p.[Arg216fs(;)Val95Met]	1
c.[645delT(;)59T>C]	p.[Arg216fs(;)Ile20Thr]	1
c.[94C>A(;)19C>T]	p.[Arg32Ser(;)Gln7Ter]	1
c.[94C>A(;)94C>A]	p.[Arg32Ser(;)Arg32Ser]	1
c.[109G>A(;)617A>G]	p.[Val37Ile(;)Asn206Ser]	1
c.[167delT(;)232dupG]	p.[Leu56fs(;)Ala78fs]	1
c.[19C>T(;)283G>A]	p.[Gln7Ter(;)Val95Met]	1
HETEROZYGOUS GENOTYPES		PROBANDS
c.[283G>A];[=]	p.[Val95Met];[=]	1
c.[29T>C];[=]	p.[Leu10Pro];[=]	1
c.[35delG];[=]	p.[Gly12fs];[=]	2
c.[35G>T];[=]	p.[Gly12Val];[=]	1
c.[59T>C];[=]	p.[Ile20Thr];[=]	1
c.[94C>A];[=]	p.[Arg32Ser];[=]	1
WILDE TYPE		PROBANDS
WT	WT	83

Genotypes were annotated according to HGVS (http://www.hgvs.org/).

TableS5. Causative genetic variants identified via WGS. Transcript NM_016239.3 was used for the *MYO15A*.

Family ID	Gene	OMIM#	Associated Phenotype	OMIM#	Zygosity	cDNA	Protein	Reference	gnomAD	CADD	GERP RS	Mutation Taster	SIFT	ClinVar	ACMG
2641	*MYO15A*	602666	Deafness, autosomal recessive 3	600316	HM	c.3757-2A>G	-	This study	NA	28.9	4.76	DC	NA	NA	LP
2726	*MYO15A*	602666	Deafness, autosomal recessive 3	600316	HT	c.843C>A	p.(Tyr281*)	This study	NA	34	2.5899	DC	NA	NA	LP
		602666	Deafness, autosomal recessive 3	600316	HT	c.8798delT	p.(Phe2933Serfs*101)	This study	NA	35	3.65	NA	NA	NA	LP

HM: Homozygous, **HT**: Heterozygous, **DC**: Disease Causing, **NA**: Not Available, **LP**: Likely Pathogenic, **VUS**: Variant of Uncertain Significance, **ACMG**: American College of Medical Genetics Guideline (Richards et al., 2015).
